# The association of socioeconomic disadvantage with postoperative complications after major elective cardiovascular surgery 

**DOI:** 10.1136/jech.2007.067470

**Published:** 2008-09-08

**Authors:** N Agabiti, G Cesaroni, S Picciotto, L Bisanti, N Caranci, G Costa, F Forastiere, C Marinacci, P Pandolfi, A Russo, C A Perucci, on behalf of the Italian Study Group on Inequalities in Health Care

**Affiliations:** 1 Epidemiology Department, Local Health Authority Rome E, Rome, Italy; 2 Epidemiology Department, Harvard School of Public Health, Boston MA, USA; 3 Epidemiology Unit, Local Health Authority, Milan, Italy; 4 Regional Health Service, Emilia Romagna, Bologna, Italy; 5 Epidemiology Unit, Piedmont Region, Turin, Italy; 6 Epidemiology Unit, Local Health Authority, La Spezia, Italy; 7 Epidemiology Unit, Local health Authority, Bologna, Italy

## Abstract

**Background:**

Understanding the mechanism by which both patient- and hospital level factors act in generating disparities has important implications for clinicians and policy-makers.

**Objective:**

To measure the association between socioeconomic position (SEP) and postoperative complications after major elective cardiovascular procedures.

**Design:**

Multicity hospital-based study.

**Subjects:**

Using Hospital Discharge Registries (ICD-9-CM codes), 19 310 patients were identified undergoing five cardiovascular operations (coronary artery bypass grafting (CABG), valve replacement, carotid endarterectomy, major vascular bypass, repair of unruptured abdominal aorta aneurysm (AAA repair)) in four Italian cities, 1997–2000.

**Measures:**

For each patient, a five-level median income index by census block of residence was calculated. In-hospital 30-day mortality, cardiovascular complications (CCs) and non-cardiovascular complications (NCCs) were the outcomes. Odds ratios (ORs) were estimated with multilevel logistic regression adjusting for city of residence, gender, age and comorbidities taking into account hospital and individual dependencies.

**Main results:**

In-hospital 30-day mortality varied by type of surgery (CABG 3.7%, valve replacement 5.7%, carotid endarterectomy 0.9%, major vascular bypass 8.8%, AAA repair 4.0%). Disadvantaged people were more likely to die after CABG (lowest vs highest income OR 1.93, p trend 0.023). For other surgeries, the relationship between SEP and mortality was less clear. For cardiac surgery, SEP differences in mortality were higher for publicly funded patients in low-volume hospitals (lowest vs highest income OR 3.90, p trend 0.039) than for privately funded patients (OR 1.46, p trend 0.444); however, the difference in the SEP gradients was not statistically significant.

**Conclusions:**

Disadvantaged people seem particularly vulnerable to mortality after cardiovascular surgery. Efforts are needed to identify structural factors that may enlarge SEP disparities within hospitals.

Socioeconomic disadvantage is associated with higher prevalence of cardiovascular risk factors,^1^ ^2^ morbidity and mortality from cardiovascular disease,^3^ ^4^ reduced access to specialist care,^5^ ^6^ and less uptake of appropriate treatment and procedures.^7^ ^8^ Incidence of postoperative complications after coronary artery bypass graft surgery (CABG)—an effective treatment for severe ischaemic heart disease—is higher in deprived patients.^9^ ^10^ Some evidence exists on association between race/ethnicity and worse prognosis after valve surgery and other cardiovascular surgery.^11^^–^13 To our knowledge no studies have explicitly investigated the influence of socioeconomic position (SEP) on postoperative complications after cardiovascular operations other than CABG in contexts outside the USA.

The relative contributions of patient and hospital factors may have important implications for addressing social disparities in health outcomes.^14^ Racial minorities are more likely to be treated by lower quality providers for CABG and for other surgeries.^15^ ^16^ In the USA uninsured people and black people are less likely to receive care for complex surgery at high-volume hospitals, where hospital volume is accepted as a structural proxy for quality.^17^ ^18^ On the other hand, the SEP gradient for hospital mortality among elective surgery patients treated in intensive care units (ICUs) was partially explained by diagnostic delays and severe comorbidity although no evidence of access to lower quality ICUs emerged.^19^

We aimed to evaluate the extent to which SEP is associated with the occurrence of postoperative complications after major elective cardiovascular procedures and to determine whether any association differs by type of surgery. Among cardiac surgery patients, we also examined whether hospital structural characteristics act as effect modifiers of the association.

## METHODS

### Source of data and cohort selection

We examined hospital records of residents in four Italian cities, Rome, Milan, Turin and Bologna, between 1997 and 2000. Discharge abstracts are routinely collected by Regional Information Systems and contain patient sociodemographic data, including census block (CB) of residence, date of admission and discharge, up to six discharge diagnoses (International Classification of Disease, 9th revision, Clinical Modification (ICD-9-CM)), up to six hospital procedures (ICD-9-CM), two of them with date, type of discharge (alive, dead, transferred to other hospital).

In order to define single episodes of care, we traced patients who were transferred to other hospitals and assessed patient discharge status at the end of the episode. We considered all records between 1 July 1997 and 30 September 2000 of patients aged 45–99 years undergoing isolated cardiovascular operations (CABG, valve replacement, carotid endarterectomy, major vascular bypass, and repair of unruptured abdominal aorta aneurysm (AAA repair)). Each condition has been selected on the basis of specific ICD-9-CM procedure codes resulting in five separate cohorts. For the AAA repair cohort, we excluded those patients a with a diagnosis of aortic aneurysm dissection. Few patients (3.5% of the total) with multiple episodes were represented multiple times. Details and codes are reported in the web-only Appendix.

### Comorbidities

Following the enhanced Elixhauser AHRQ-Web-ICD-9-CM coding algorithm,^20^ we defined eight selected comorbidities that can play a role in the outcomes of surgery: cardiac and circulatory disease, vascular disease (including cerebrovascular), hypertension, pulmonary disease, renal disease, liver disease, tumours. For each surgery, we did not consider as comorbidity the diagnosis that could reflect the primary surgical indication. We identified conditions on the basis of ICD-9-CM codes registered in hospital admissions in the previous 6 months. Details and codes are reported in the web-only Appendix.

### Socioeconomic position

We used a small area income index based on the census block (CB) of residence, developed by the “Italian Study Group on Inequalities in Health Care”, and already used in other settings.^21^ ^22^ Briefly, the cities were divided into CBs with the mean number of inhabitants per CB ranging from 200 in Bologna to 500 in Rome. A record linkage between the 1998 Tax Register and the Population Registers connected family status information to income data for each subject, then the net family income and equivalised per capita income were calculated. Data were aggregated at the CB level, and the median value for each CB was obtained; quintiles of the income distribution by CB were calculated for each city (I, high income; V, low income). Using the CB at discharge, the index was attributed to all individuals under study.

### Hospital level measures

For CABG and valve cohorts, we studied two organisational characteristics of hospital services: hospital payment structure and volume of surgery.

In Italy universal access and comprehensive coverage exist in a publicly funded health system.^23^ According to payment structure of the hospital where the intervention was performed, hospitals were classified as follows: (1) public, referring to publicly financed hospital care; (2) private, referring to private care provision. The first group includes both public and private hospitals with full-cost reimbursement and the second group includes all hospitals in which patients must pay (partially or fully).

In the public group we also distinguished two categories of procedure volume for cardiac surgery: (1a) public low volume, (1b) public: others. We determined hospital procedure volumes by calculating the total number of specific procedures in our sample for each hospital in the 4-year study period. According to their volume, we contrasted those hospitals under the lowest 20th percentile of distribution (public low volume) with all others (public: others). The procedures cut-off points were 50 per year for CABG, and 43 per year for valve replacement.

### Outcome measures

The main outcome was in-hospital mortality within 30 days after the intervention. Less than 2% of all records (with the exception of 4% for endarterectomy) were missing the date of major intervention. In those cases, we calculated mortality within 30 days from the date of admission plus the mean waiting time for the selected procedure.

We defined two groups of complications as secondary outcomes: (1) cardiovascular complications (CCs) including acute myocardial infarction, arrhythmia, cardiogenic shock, cardiac arrest, cerebrovascular complications, arterial acute diseases, and (2) non-cardiovascular complications (NCCs) including complications of anaesthesia, decubitus ulcer, sepsis, deep vein thrombosis/pulmonary embolism, foreign body left during procedure, selected infections due to medical care, pneumonia, postoperative haemorrhage or haematoma, postoperative physiological and metabolic derangement, postoperative wound dehiscence, transfusion reaction, gastrointestinal complications. We identified these conditions by ICD-9-CM codes in the index (any position) or in any potential subsequent admission within 30 days after surgery (main diagnosis) on the basis of the coding algorithm for Patient Safety Indicators recently developed by the US Agency for Health Care Research and Quality.^24^ Definitions and details on ICD-9-CM codes are reported in the web-only Appendix.

### Statistical analysis

To examine the associations between income and outcomes, we estimated odds ratios (OR) separately for the five cohorts. In order to take into account the hierarchical structure of the data, we performed multilevel modelling using mixed logit models with random intercepts for hospitals and individuals and fixed intercepts for cities of residence. Individual covariates were age (linear), gender, comorbidities and income. Backward stepwise procedures were used to discard those variables that were not associated with the specific outcome (p>0.20). Quintiles of income were considered as a categorical variable using the first quintile (high income) as the reference group, and p values for linear trend were calculated using the Wald test.

Unobserved hospital level factors affecting treatment can be related to patient characteristics.^25^ We could not exclude the possibility that disadvantaged people in our sample were systematically admitted to hospitals that provided low quality of care. Therefore, as an additional analysis, we performed a single-level logistic model with a fixed effect for hospital (with dummy variables for all hospitals) and all the other covariates. In that way, we tried to isolate the “within-hospital” SEP effect on the outcomes. In this case, we adjusted estimates of variance for clustering and used robust estimates.

Finally, we hypothesised that the income/mortality relationship may vary according to hospital structural characteristics. Therefore, to further clarify the role of hospital level factors, we tested the association between income and mortality among those with CABG and valve replacement stratifying by organisational characteristics of hospital services. Effect modification was tested using an interaction term in the regression model and the likelihood ratio test.

A sensitivity analysis was performed for the city of Rome to evaluate whether the results would be different using as outcome 30-day mortality regardless of the place of death, in case of an association between income and place of death. We obtained vital status-linking records to the regional mortality information system. In addition, a final sensitivity analysis was conducted excluding all those patients with repeated episodes of care.

Datasets were prepared using ORACLE Database 10g, and all statistical analysis was performed using the software STATA version 10. All tests of significance are at the 5% level, and all p values reported are two sided.

## RESULTS

### Characteristics of patients by type of surgery and by SEP

The distribution of surgeries in the cities reflects the number of inhabitants (table 1). The largest cohort was CABG and the smallest was major vascular bypass. Except for valve replacement, the cardiovascular operations were more common among men. Among those with valve replacement and CABG there was a higher prevalence of young people, whereas the opposite held among those with carotid endarterectomy and AAA repair. In all the cohorts—with the exception of AAA repair—the proportion of people in the lowest income levels was higher than 20%. The prevalence of comorbidities and the incidence of outcomes varied widely across the cohorts.

**Table 1:** Characteristics of patients by type of surgery, four Italian cities, 1998–2000

	Coronary artery bypass grafting	Valve replacement	Carotid endarterectomy	Major vascular bypass	Repair of unruptured abdominal aorta aneurysm
Discharges	7948	2770	5427	1200	2638
Subjects	7930	2733	4843	1175	2629
Subjects with multiple discharges	18	36	582	23	9
City of residence					
Bologna	584	276	599	79	251
Milan	2907	942	1709	374	980
Rome	3015	990	2470	573	1029
Turin	1442	562	649	174	378
Sociodemographic characteristics (%)					
Women	18.7	53.0	33.8	16.8	8.5
45–59 years age	24.1	25.7	8.0	15.3	6.1
75+ years age	14.0	18.3	31.7	22.3	31.6
Area-based income index (quintiles)					
I high	18.2	16.9	15.5	14.8	20.9
II	19.4	18.1	17.9	16.1	20.1
III	20.1	21.4	19.8	20.9	20.2
IV	20.9	21.7	22.6	21.4	20.8
V low	21.5	21.8	24.2	26.8	18.0
Comorbidities (%)					
Cardiac and circulatory disease	13.1	30.3	12.9	11.9	11.8
Vascular disease including cerebrovascular	6.8	5.8	9.8	10.8	6.6
Hypertension	24.1	9.8	17.1	11.4	10.8
Pulmonary disease	2.7	4.1	3.3	6.8	4.7
Diabetes	12.1	3.5	8.1	6.8	2.2
Renal disease	1.5	1.5	1.2	2.0	2.2
Liver disease	0.5	1.2	0.4	0.6	0.5
Tumours	0.7	0.9	1.0	2.2	2.2
Organisational characteristics of hospital care (%)					
Public: high volume	63.5	63.8	–	–	–
Public: low volume	14.3	15.4	–	–	–
Private	22.2	20.8	–	–	–
Outcomes (%)					
In-hospital 30-day mortality	3.7	5.7	0.9	8.8	4.0
Cardiovascular complications	5.1	6.4	5.3	11.8	5.3
Non-cardiovascular complications	5.4	7.1	2.1	13.8	9.2

**What is already known on this subject**

- Disparity in health outcomes is a component of the complex picture of inequity in health.
- Disadvantaged people tend to experience worse outcomes after care even in countries with universal coverage.
- Few studies have examined the role of hospital level factors on social disparities in outcomes after surgery, and those available have mainly evaluated race/ethnicity.

**What this study adds**

- Poor people who undergo cardiac surgery are more likely to die after operation than their rich counterparts in Italy; for vascular surgery, a relationship between socioeconomic position (SEP) and outcomes is also suggested.
- The association between SEP and outcomes after cardiac surgery is particularly evident in low-volume public hospitals whereas differences narrow in private hospitals.
- Interventions aimed at improving the quality of surgical care—especially where the care is suboptimal—are recommended as a means to reduce health disparities.

Lower income was associated with higher proportions of women (with exception of major vascular bypass) and of younger people (with the exception of AAA repair) (table 2). The prevalence of comorbidities was generally higher in low-income groups, particularly among cardiac patients.

**Table 2:** Characteristics of patients by income level for the five different surgeries

	Area-based income index (quintiles)				
	I high	II	III	IV	V low
Coronary artery bypass grafting					
Discharges (n)	1445	1538	1594	1660	1711
Women (%)	13.6	16.3	18.5	19.6	24.3
45–59 years (%)	22.4	25.6	25.4	22.6	24.3
75+ years (%)	17.4	13.2	14.5	13.7	11.4
Hypertension (%)	21.3	24.2	24.5	24.8	25.3
Pulmonary disease (%)	2.4	2.0	2.6	2.6	3.6
Diabetes (%)	8.4	11.6	11.0	14.0	14.7
Valve replacement					
Discharges (n)	469	502	592	602	605
Women (%)	47.3	49.8	53.9	55.1	57.2
45–59 years age (%)	24.3	28.1	21.8	25.3	29.1
75+ years age (%)	20.0	17.7	18.1	17.8	18.2
Hypertension (%)	8.5	8.6	9.6	9.8	11.9
Pulmonary disease (%)	2.8	3.6	4.7	3.7	5.3
Diabetes (%)	2.4	1.8	3.7	4.2	5.0
Carotid endarterectomy					
Discharges (n)	840	971	1074	1226	1316
Women (%)	29.9	33.1	33.3	33.0	37.9
45–59 years (%)	8.0	8.9	6.6	8.0	8.4
75+ years (%)	36.0	32.1	31.0	31.1	29.7
Hypertension (%)	13.6	16.7	18.2	17.5	18.5
Pulmonary disease (%)	2.6	2.5	2.5	3.8	4.6
Diabetes (%)	6.7	7.5	8.9	8.7	8.2
Major vascular bypass					
Discharges (n)	178	193	251	257	321
Women (%)	17.4	16.6	16.7	16.0	17.1
45–59 years (%)	21.4	13.5	7.6	14.4	19.6
75+ years (%)	32.6	27.5	23.9	17.9	15.6
Hypertension (%)	9.0	10.4	13.6	12.5	10.9
Pulmonary disease (%)	6.7	6.2	5.6	8.2	6.9
Diabetes (%)	5.1	6.2	8.0	8.6	5.6
Repair of unruptured abdominal aorta aneurysm					
Discharges (n)	552	530	534	548	474
Women (%)	8.7	8.5	6.2	8.4	11.0
45–59 years (%)	7.3	6.8	4.9	5.5	6.1
75+ years (%)	32.1	30.4	33.2	28.1	34.8
Hypertension (%)	10.1	12.5	10.3	10.4	10.8
Pulmonary disease (%)	5.3	4.7	4.1	3.5	6.1
Diabetes (%)	1.3	2.5	3.0	2.0	2.3

### Association between SEP and outcomes after surgery

For CABG there was evidence of an association between income and mortality (OR 1.93 lowest vs highest income; p trend 0.023). For valve replacement, such an association was suggested (OR 1.65, p trend 0.090). In the AAA repair cohort, an increase in the risk of mortality was observed (OR 2.03), although the trend was not statistically significant. No significant associations were found for carotid endarterectomy and major vascular bypass. When we adjusted for the hospital effect using a single-level analysis with a fixed effect for hospitals, the associations between income and mortality persisted (for CABG, OR 1.69, p value 0.046) indicating that the estimated SEP disparity reflects within-hospital differences among low- and high-income patients (table 3).

**Table 3:** Association between income and in-hospital 30-day mortality after elective cardiovascular surgery

	Area-based income index (quintiles)					p Trend
	I high	II	III	IV	V low	
*Coronary artery bypass grafting*						
%	2.8	3.5	4.0	2.7	5.1	
OR* (95% CI)	1.00	1.21 (0.77 to 1.91)	1.42 (0.91 to 2.22)	0.89 (0.55 to 1.43)	1.93 (1.23 to 3.05)	0.023
OR† (95% CI)	1.00	1.13 (0.74 to 1.72)	1.26 (0.84 to 1.89)	0.84 (0.54 to 1.30)	1.69 (1.15 to 2.48)	0.046
Valve replacement						
%	4.3	5.0	6.1	6.2	6.5	
OR* (95% CI)	1.00	1.11 (0.56 to 2.20)	1.34 (0.70 to 2.56)	1.41 (0.74 to 2.69)	1.65 (0.86 to 3.18)	0.090
OR† (95% CI)	1.00	1.08 (0.57 to 2.05)	1.28 (0.70 to 2.36)	1.30 (0.72 to 2.37)	1.52 (0.83 to 2.77)	0.126
Carotid endarterectomy						
%	0.6	1.4	1.3	0.5	0.5	
OR* (95% CI)	1.00	2.76 (0.98 to 7.75)	2.46 (0.87 to 6.91)	0.87 (0.26 to 2.88)	0.97 (0.30 to 3.10)	0.180
OR† (95% CI)	1.00	3.01 (0.96 to 9.50)	2.61 (0.84 to 8.09)	0.69 (0.18 to 2.59)	0.96 (0.27 to 3.43)	0.066
Major vascular bypass						
%	11.2	9.3	10.4	7.4	6.9	
OR* (95% CI)	1.00	0.38 (0.05 to 3.21)	0.71 (0.11 to 4.73)	0.35 (0.05 to 2.72)	0.51 (0.08 to 3.47)	0.553
OR† (95% CI)	1.00	0.60 (0.26 to 1.37)	0.81 (0.37 to 1.76)	0.49 (0.21 to 1.12)	0.58 (0.26 to 1.29)	0.203
*Repair of unruptured abdominal aorta aneurysm*						
%	2.7	5.1	3.8	3.7	5.1	
OR* (95% CI)	1.00	1.98 (1.03 to 3.80)	1.40 (0.70 to 2.80)	1.48 (0.74 to 2.95)	2.03 (1.03 to 3.97)	0.168
OR† (95% CI)	1.00	1.81 (0.90 to 3.62)	1.24 (0.61 to 2.55)	1.22 (0.58 to 2.58)	1.68 (0.82 to 3.43)	0.493

*OR, two-level logistic regression (hospital and subject). OR adjusted for city of residence, gender, age and comorbidities.

†OR single level logistic regression. OR adjusted for city of residence, gender, age, comorbidities and hospital.

There was no evidence of statistically significant association between income and CCs and NCCs in any cohort (table 4).

**Table 4:** Association between income and outcomes after elective cardiovascular surgery

	Cardiovascular complications						Non-cardiovascular complications					
	Area-based income index (quintiles)						Area-based income index (quintiles)					
	I high	II	III	IV	V low	p Trend	I high	II	III	IV	V low	p Trend
*Coronary artery bypass grafting*												
%	5.2	4.7	5.4	5.2	6.4		5.8	6.6	5.7	5.1	5.7	
OR^1^ (95% CI)	1.00	0.91 (0.71 to 1.47)	1.02 (0.67 to 1.38)	0.96 (0.85 to 1.72)	1.21 (0.96 to 1.69)	0.249	1.00	1.14 (0.81 to 1.61)	0.99 (0.70 to 1.41)	0.90 (0.63 to 1.28)	1.02 (0.72 to 1.44)	0.594
OR^2^ (95% CI)	1.00	0.87 (0.62 to 1.23)	0.93 (0.67 to 1.30)	0.89 (0.64 to 1.24)	1.08 (0.79 to 1.49)	0.543	1.00	1.11 (0.81 to 1.53)	0.97 (0.70 to 1.33)	0.88 (0.63 to 1.22)	0.99 (0.71 to 1.36)	0.455
*Valve replacement*												
%	6.1	6.6	5.7	6.4	9.0		7.1	8.0	6.7	8.3	8.2	
OR* (95% CI)	1.00	1.11 (0.64 to 1.91)	0.93 (0.54 to 1.59)	1.02 (0.60 to 1.72)	1.54 (0.94 to 2.53)	0.114	1.00	1.10 (0.63 to 1.91)	0.93 (0.54 to 1.61)	1.19 (0.70 to 2.02)	1.26 (0.73 to 2.16)	0.352
OR† (95% CI)	1.00	1.09 (0.63 to 1.88)	0.88 (0.51 to 1.51)	0.97 (0.57 to 1.65)	1.47 (0.89 to 2.42)	0.190	1.00	1.03 (0.62 to 1.72)	0.89 (0.54 to 1.47)	1.13 (0.70 to 1.83)	1.18 (0.72 to 1.92)	0.421
*Carotid endarterectomy*												
%	5.1	5.2	6.2	5.8	5.6		2.4	2.1	2.0	2.1	2.3	
OR* (95% CI)	1.00	0.96 (0.58 to 1.59)	1.23 (0.76 to 2.00)	1.07 (0.66 to 1.72)	1.00 (0.62 to 1.61)	0.924	1.00	0.87 (0.39 to 1.95)	0.86 (0.39 to 1.89)	0.84 (0.39 to 1.81)	1.02 (0.49 to 2.14)	0.928
OR† (95% CI)	1.00	0.87 (0.56 to 1.36)	1.09 (0.71 to 1.66)	0.93 (0.61 to 1.42)	0.88 (0.58 to 1.33)	0.634	1.00	0.98 (0.51 to 1.85)	0.95 (0.50 to 1.79)	0.93 (0.50 to 1.72)	0.99 (0.54 to 1.82)	0.956
*Major vascular bypass*												
%	16.3	10.3	12.1	15.8	12.6		23.6	10.3	20.7	14.7	13.4	
OR* (95% CI)	1.00	0.22 (0.03 to 1.39)	0.36 (0.07 to 1.89)	1.26 (0.25 to 6.29)	0.79 (0.17 to 3.73)	0.426	1.00	0.37 (0.14 to 0.95)	0.88 (0.43 to 1.79)	0.61 (0.28 to 1.31)	0.54 (0.25 to 1.19)	0.325
OR† (95% CI)	1.00	0.57 (0.28 to 1.14)	0.59 (0.31 to 1.13)	0.99 (0.52 to 1.89)	0.84 (0.44 to 1.59)	0.613	1.00	0.36 (0.17 to 0.74)	0.80 (0.45 to 1.43)	0.56 (0.30 to 1.02)	0.58 (0.33 to 1.01)	0.275
*Repair of unruptured abdominal aorta aneurysm*												
%	4.3	5.8	4.9	6.0	7.0		10.6	9.1	9.7	12.1	9.0	
OR* (95% CI)	1.00	1.33 (0.75 to 2.35)	1.10 (0.61 to 1.99)	1.36 (0.77 to 2.39)	1.56 (0.88 to 2.75)	0.159	1.00	0.83 (0.54 to 1.29)	0.92 (0.60 to 1.42)	1.07 (0.70 to 1.62)	0.78 (0.49 to 1.22)	0.674
OR† (95% CI)	1.00	1.20 (0.65 to 2.22)	1.01 (0.54 to 1.89)	1.16 (0.63 to 2.12)	1.38 (0.76 to 2.49)	0.368	1.00	0.78 (0.50 to 1.22)	0.88 (0.57 to 1.36)	0.96 (0.63 to 1.47)	0.72 (0.44 to 1.15)	0.415

*OR two-level logistic regression (hospital and subject). OR adjusted for city of residence, gender, age and comorbidities.

†OR single-level logistic regression. OR adjusted for city of residence, gender, age, comorbidities and hospital.

Cardiac patients were treated in 27 public low-volume (procedure volume range in the 4-year period: CABG 1–194, valve 1–65); 10 public: others (procedure volume range: CABG 235–726, valve 133–319) and 8 private hospitals. Mortality tended to be lower in private than in public hospitals (2.6% vs 4.6%, OR 0.54, 95% CI 0.40 to 0.71 adjusted for city of residence, age, gender and comorbidities). The income/mortality association was stronger among cardiac patients in public hospitals (lowest vs highest income OR 1.65, p trend 0.022) in comparison with those in private care (OR 1.46, p trend 0.444) (table 5). However, a formal test for interaction was not statistically significant (p = 0.465). This association was even higher for those patients treated in public low-volume hospitals (OR 3.90, p trend 0.039). Again, a formal test for interaction was not statistically significant (p = 0.230).

**Table 5:** Association between income and in-hospital 30-day mortality after CABG and valve replacement, by organisational characteristics of hospital care

	Area-based income index (quintiles)					p Trend
	I high	II	III	IV	V low	
Public: low volume						
n (% death)	282 (2.1)	280 (4.3)	323 (3.4)	333 (3.3)	345 (6.1)	
OR (95% CI)	1.00	2.57 (0.83 to 7.95)	1.71 (0.56 to 5.22)	1.79 (0.59 to 5.40)	3.90 (1.23 to 12.4)	0.039
Public: high volume						
n (% death)	1042 (4.4)	1300 (4.1)	1.394 (5.4)	1.497 (3.9)	1.584 (6.0)	
OR (95% CI)	1.00	0.87 (0.55 to 1.37)	1.17 (0.76 to 1.80)	0.80 (0.51 to 1.25)	1.39 (0.92 to 2.12)	0.131
Overall public						
n (% death)	1324 (3.9)	1.580 (4.1)	1.717 (5.0)	1.830 (3.8)	1.929 (6.0)	
OR (95% CI)	1.00	1.04 (0.69 to 1.58)	1.26 (0.85 to 1.88)	0.91 (0.60 to 1.37)	1.65 (1.12 to 2.43)	0.022
Private						
n (% death)	590 (1.5)	460 (2.8)	469 (3.0)	432 (3.0)	387 (2.8)	
OR (95%CI)	1.00	1.75 (0.73 to 4.21)	1.66 (0.70 to 3.96)	1.83 (0.75 to 4.43)	1.46 (0.57 to 3.72)	0.444

Two-level logistic regression (hospital and subject). OR adjusted for city of residence, gender, age and comorbidities.

In all analyses, comorbidities seemed not to confound the associations. In the sensitivity analysis conducted on the data set of Rome, similar results were found using 30 day mortality regardless of the place of death. Finally, very similar results were obtained when we excluded those patients with multiple episodes.

## DISCUSSION

This study describes the occurrence of postoperative complications after major cardiovascular surgery in a large multicity Italian sample. Socioeconomic disadvantage is associated with worse outcomes after cardiac surgery, but no clear indication of a similar relationship for vascular surgery has been found. Disparities in outcomes after cardiac surgery are strongest in public low-volume hospitals.

There is still little research on the relationship between SEP and healthcare outcomes and the results are inconsistent.^3^ ^10^ ^21^ ^26^ ^27^ Our study confirms previous results for CABG and contributes to the knowledge on other types of cardiovascular procedures.^7^ ^9^ ^10^ Higher deprivation score has been associated with younger age, more comorbidities, and more postoperative cardiovascular complications after CABG.^9^ ^18^ It is of note that the majority of available studies have focused on racial disparities among patients over 65 years in the USA, whereas less information is available on other indicators of social status.^28^^–^33

It has been suggested that disadvantaged people who underwent surgery might have higher baseline risks than well-off patients, resulting in worse prognosis.^3^ ^7^ ^10^ ^18^ ^19^ The greater presence of comorbid conditions in low-income people in our study supports this hypothesis. Lack of knowledge about the procedure and its benefits might be higher among social disadvantaged groups. In the USA black people tend to postpone elective surgery and experience more advanced disease.^10^ ^19^ In our study, evidence of disparity in outcomes is less clear for procedures other than CABG; although results are not statistically significant, possible associations cannot be excluded. The reason is difficult to explain. Heterogeneous SEP differences in the prevalence of comorbidities among the different cohorts support the hypothesis that poor people undergoing cardiac surgery had worse baseline conditions than poor people with vascular surgery in comparison with their respective rich counterparts. For that reason low SEP patients who had cardiac surgery would have been more susceptible to complications than low SEP vascular patients. Alternatively, cardiac surgery itself may present more dangers to those who are vulnerable than does vascular surgery. On the other hand, cardiac and vascular surgery tend to share many processes of care related to anaesthesia, intensive care and postoperative care. Finally, unknown individual (ie, perioperative risk) or system factors (ie, surgeon’s specialty or skill) that we were not able to measure in this study might have contributed to our findings.

Few studies have examined the role of hospital level factors on social disparities in health outcomes, and those available have mainly evaluated race/ethnicity. Higher rates of surgical complications among black people are largely explained by differential use of high-quality hospitals.^13^^–^18 However, a large proportion of racial differences in post-procedure mortality has been found to be unrelated to hospital volume, an accepted marker of quality.^13^ ^34^ ^35^ Similarly, differences in health outcomes after acute myocardial infarction according to SEP have been found to be mainly explained by cardiovascular risk factors than by access to appropriate care, adding more to the debate on mechanisms of disparities.^27^ We considered both patient and hospital level characteristics by applying recently developed methods^18^ ^25^ ^27^ and we suggest that hospital structural factors (in our case public low-volume hospitals) may act as effect modifiers. Better organisation and processes of care, more skilled surgeons and adequate numbers of nurses in private facilities could be partially responsible for the observed homogeneous outcomes across social groups. However, the lower mortality among those treated in private than in public hospitals in our study suggests less severe preoperative status, the main risk factor for outcome after surgery.^12^ ^36^ Worse preoperative status among patients in public hospitals could be related to the shorter waiting times for surgery in privately funded hospitals.^37^ Its different distribution among patients in public and private hospitals could partially explain the results.

The population-based design, the number of operations and the validated algorithms are the strengths of this study. Our study is one of the first studies in Europe to test the feasibility of AHRQ ICD-9-CM-based surgical care indicators, although administrative datasets have been previously used to identify postoperative complications.^38^ Despite their wide use as a valuable source for healthcare research, hospital discharge data have several limitations, which have been repeatedly recognised.^39^ In our case, the datasets do not include information on relevant prognostic factors such as operative priority. Under-reporting is also possible, as proven by a validation study of CABG patients in Rome.^40^ However, it is unlikely that different reporting across hospitals and misclassification errors of comorbidity or complications are associated with SEP. It is more probable, in contrast, that true incidence of complications and their severity may be higher than reported, weakening the evidence for existing socioeconomic disparities. Different comorbidity measurements based on ICD-9-CM or ICD-10-CM code have been tested in cardiovascular research despite their lower ability in predicting outcomes in comparison to clinical data;^41^ ^42^ the Elixhauser method allows a comprehensive definition of comorbidity and has proven superior to others in predicting mortality after selected conditions.^43^

Some limitations should be underlined. First, we are concerned that our measure of volume might not accurately reflect experience with the procedure because it is based on the studied cohorts. It is likely, however, that our surrogate measure is well related to the true procedure volume. Second, attributing an aggregated indicator may not accurately represent the individual’s true SEP, and the association may be distorted.^44^ Our area-based income index includes economic resources provided by work, pension, real estate and investments which contribute to defining the material well-being and standards of living of all family members. However, income might not be a good marker for other social conditions relevant for health, and risk factors such as occupation or level of education.^45^ Although we did not have information on other important social determinants of health, other studies have shown that even after adjustment for such variables, the association between economic resources and health persists.^46^ Moreover, at the small area level, the predictive power of economic poverty indicators has been showed to be comparable with that related to composite SEP indices.^47^ We observed a correlation coefficient higher than 0.7—even among elderly people—between the income index and more composite SEP indicators available both for Rome and Turin.^48^ These results made us confident in using the income indicator as a good proxy for the complex construct of SEP.

In conclusion, this study found that SEP influences postoperative status after selected major cardiovascular surgery in the Italian hospital care system, corroborating the evidence from other countries. Low SEP persons undergoing surgical procedures may be more vulnerable to adverse events and should be monitored carefully. Explicit efforts should be made to identify systemic factors that amplify inequities.
